# SNR analysis: molecular investigation of an anthrax epidemic

**DOI:** 10.1186/1746-6148-6-11

**Published:** 2010-02-28

**Authors:** Giuliano Garofolo, Andrea Ciammaruconi, Antonio Fasanella, Silvia Scasciamacchia, Rosanna Adone, Valentina Pittiglio, Florigio Lista

**Affiliations:** 1 Istituto Zooprofilattico Sperimentale della Puglia e della Basilicata, Anthrax Reference Institute of Italy- Foggia, Italy; 2 Army Medical and Veterinary Research Institute - Rome, Italy; 3 Istituto Superiore di Sanità - Rome, Italy

## Abstract

**Background:**

In Italy, anthrax is endemic but occurs sporadically. During the summer of 2004, in the Pollino National Park, Basilicata, Southern Italy, an anthrax epidemic consisting of 41 outbreaks occurred; it claimed the lives of 124 animals belonging to different mammal species. This study is a retrospective molecular epidemiological investigation carried out on 53 isolates collected during the epidemic. A 25-loci Multiple Locus VNTR Analysis (MLVA) MLVA was initially performed to define genetic relationships, followed by an investigation of genetic diversity between epidemic strains through Single Nucleotide Repeat (SNR) analysis.

**Results:**

53 *Bacillus anthracis *strains were isolated. The 25-loci MLVA analysis identified all of them as belonging to a single genotype, while the SNR analysis was able to detect the existence of five subgenotypes (SGTs), allowing a detailed epidemic investigation. SGT-1 was the most frequent (46/53); SGTs 2 (4/53), 3 (1/53) 4 (1/53) and 5 (1/53) were detected in the remaining seven isolates.

**Conclusions:**

The analysis revealed the prevalent spread, during this epidemic, of a single anthrax clone. SGT-1 - widely distributed across the epidemic area and present throughout the period in question - may, thus, be the ancestral form. SGTs 2, 3 and 4 differed from SGT-1 at only one locus, suggesting that they could have evolved directly from the latter during the course of this epidemic. SGT-5 differed from the other SGTs at 2-3 loci. This isolate, thus, appears to be more distantly related to SGT-1 and may not be a direct descendant of the lineage responsible for the majority of cases in this epidemic. These data confirm the importance of molecular typing and subtyping methods for in-depth epidemiological analyses of anthrax epidemics.

## Background

**Figure 1:**
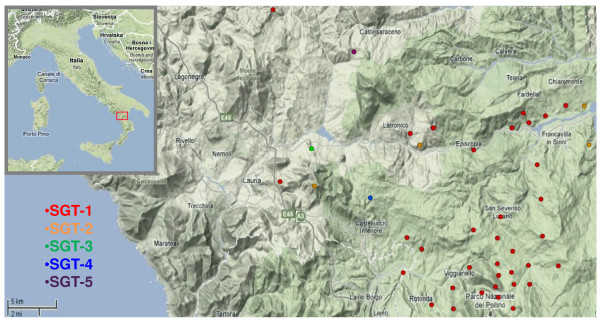
**Map of the Pollino national Park 2004 anthrax epidemic**. Geographical representation (GIS data) of the epidemic, with its 41 outbreaks. The five subgenotypes are marked in different color fonts. ^©^2009 Google - Map data ^©^2009 Tele Atlas.

In the region of Basilicata, Southern Italy, anthrax outbreaks are typically isolated, self containing, and involve unvaccinated herbivores. Epidemics are rare, and often occur when a rainy spring is followed by a dry summer [1-5]. During the spring and summer of 2004, as a result of such weather conditions in the Pollino National Park, an anthrax epidemic occurred. The affected area included 13 towns and involved 41 farms over an area of about 900 Km^2^, with a livestock population numbering about 7,000 cattle and 33,000 between sheep and goats. In 40 days, 81 cattle, 15 sheep, nine goats, eleven horses and eight red deer died (Figure 1). The anthrax epidemic evolved in three different phases. The first, counting 26 outbreaks, was the most critical. The second and third phases, with eight and six outbreaks, respectively, were less severe.

An additional outbreak preceded the epidemic by about one month [6]. Several epidemiological factors may have contributed to this phenomenon. In this, as in other endemic areas, spores resulting from previous outbreaks may remain in the soil, thus facilitating the spread of anthrax among livestock through grazing [7,8]. In addition, anthrax infected carcasses are seldom removed. These carcasses are not isolated from the wild animals populating the Pollino National Park (deer, wild boars), resulting in a persistent source of infection in the environment [2]. Furthermore, the abundance, at this time of year, of both biting (e.g. tabanid) and non biting flies, which may act as mechanical vectors, could also have contributed to the persistence of anthrax [9-14].

Genetically, *B. anthracis *is a relatively homogeneous bacteria species. Not surprisingly, then, discriminating between strains isolated from epidemiologically linked outbreaks is not an easy task [15]. Different studies typed and differentiated *B. anthracis *isolates using Single Nucleotide Polymorphisms (SNP) analysis and Multiple Locus VNTR analysis (MLVA) [16-22]. In an epidemic, however, these methodologies are not likely to find genetic variation. The Single Nucleotide Repeats (SNR) analysis described by Stratilo *et al*. increases the likelihood of differentiating closely related isolates [23]. Unfortunately, due to the presence of poly-A sequences, such polymorphisms are difficult to detect both with electorphoretic fragment analysis and with direct sequencing.

In this study, a retrospective molecular epidemiological investigation was performed, comparing 25-loci MLVA and two SNR analyses. We applied the modified SNR technique described by Kenefic et al. (KEN-MTD) as well as Stratilo's original SNR method (STR-MTD), selecting the four loci with the highest diversity indices (D = 0.57-0.90; where D = 1-Σ [allele frequency]2) [23,24]. The SNR primer panels used have two loci in common (CL33, CL12) and two distinct loci (STR-MTD: CL1, CL37) (KEN-MTD: CL10, CL35). Two different genetic analyzers (DNA sequencers) were used to verify results. This was done to address the technical difficulty in correct allele assignment.

## Results

### MLVA 25

The 25-loci MLVA analysis classified all 53 B. anthracis isolates as belonging to a single genotype within cluster A1.a, as defined by Lista et al. (Table 1) [17].

**Table 1:** Results of MLVA genotyping and SNR subgenotyping of *B. anthracis *isolates from the Pollino National Park 2004 epidemic

MLVA 25									
**Cluster**	**No. of Isolates**	**Allele Coding**							

A1.a	53	VrrA: 10; vrrB1: 16; vrrB2: 7; vrrC1: 57; vrrC2: 21; CG3: 1; bams1: 13; bams 3: 30; bams5: 7; bams13: 30; bams15: 45; bams21: 10; bams22: 16; bams23: 11; bams24: 11; bams25: 13; bams28: 14; bams30: 75; bams31: 64; bams34: 8; bams44: 8; bams51: 9; bams53: 8; pXO1: 7; pXO2: 7.							

**SNR ANALYSES**									

		**STR-MTD**				**KEN-MTD**			

**Subgenotype**	**No. of Isolates**	**Loci**				**Loci**			

		**CL33**	**CL12**	**CL1**	**CL37**	**CL33** **HM1**	**CL12** **HM2**	**CL10** **HM6**	**CL35** **HM13**

SGT-1	46	294	172	243	212	83	91	107	117

SGT-2	4	**293**	172	243	212	**82**	91	107	117

SGT-3	1	**295**	172	243	212	**84**	91	107	117

SGT-4	1	294	**173**	243	212	83	**92**	107	117

SGT-5	1	**289**	**173**	243	212	**78**	**92**	**106**	117

TOTAL	53	-	-	-	-	-	-	-	-

Table Top - The 25-loci MLVA cluster group with its allele coding data as defined by Lista et al. [17]. Bottom - SNR alleles are displayed as fragment sizes (base pairs) obtained through capillary electrophoresis. Stratilo's locus nomenclature was used throughout [23]. For results obtained with the KEN-MTD, Kenefic's reference codes are also reported [24]. Alleles differing from those of the most common subgenotype, SGT-1, are underlined and highlighted in bold.

### SNR analysis

**Figure 2:**
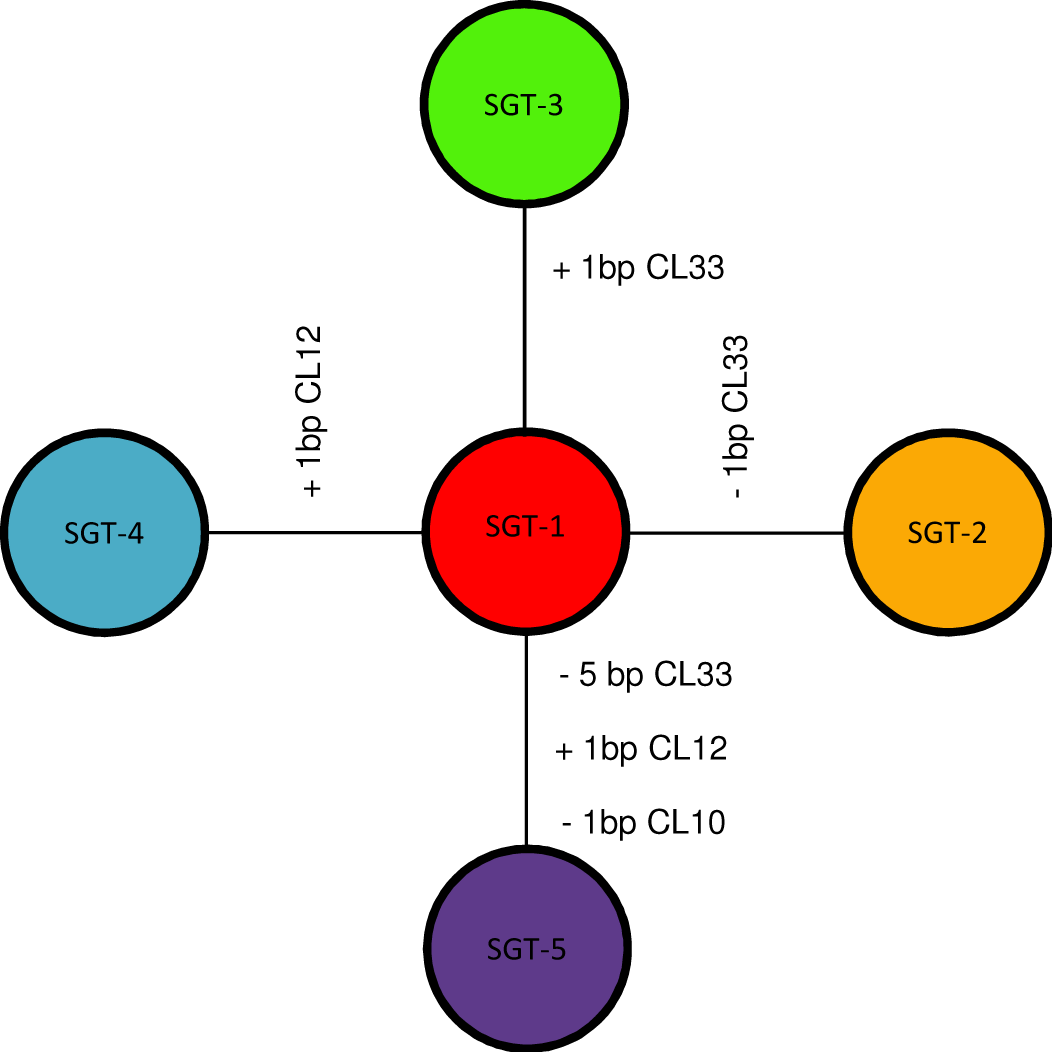
**Genetic relationships between epidemic strains sample**. The mutational steps from the dominant subgenotype to the minor subgenotypes are shown along the branches, indicating the mutated loci and the number of base pairs deleted (-) or inserted (+).

SNR analysis identified five SGTs. Of 53 isolates, 46 were classified as SGT-1, compared to which four isolates, classified as SGT-2, had a single-base pair deletion corresponding to locus CL33; SGT-3 and SGT-4, each with a single isolate, exhibited insertions in loci CL33 and CL12, respectively, while SGT-5, again with a single isolate, differed from SGT-1 at three loci, with a five-base pair deletion at CL33, an insertion into the CL12 locus, and a deletion at CL10 (Table 1; figure 2).

## Discussion

Due to homoplasy, the high mutation rate of SNR loci, estimated at 10^-4 ^per generation in *B. anthracis*, does not allow a correct definition of phylogenetic relationships between different isolates [15]. SNR is, however, able to detect genetic diversity between closely related strains as would occur in an epidemic [6]. Our method of analysis involved the initial use of 25-loci MLVA to define genetic relationships, and a subsequent investigation of genetic diversity between epidemic strains through SNR analysis.

The 25-loci MLVA assigned all strains to a single genotype within the cluster A1.a, evidence of their autochthonous origin. This particular genotype is frequently found in Basilicata. The two SNR analyses, STR-MTD and KEN-MTD, yielded the same result, identifying five SGTs. The difference between these methods in terms of the amplicon sizes obtained for the common loci is a consequence of the use of different primer pairs. We found the KEN-MTD more useful, as it allows for a multiplexed reaction and permits faster and more reliable detection of fragment sizes, owing to smaller amplicons sizes. Moreover, the KEN-MTD revealed an additional difference between SGT-5 and SGT-1, an allele in the CL10 (Table 1). Polymorphisms were only discovered among the loci with the highest diversity indices (CL33, CL12, CL10) [23].

SGT-1, the most common, was distributed across the epidemic area and present throughout the period under study. SGT-1 was also detected in soil from a grave site, and in the feces of wild boar collected in the same area, suggesting it is the probable ancestral strain. SGTs 2-4 were rarer (6/53) and not present in all phases of the epidemic. These SGTs could represent intra epidemic mutational steps descended from SGT-1. SGT-5, with three loci exhibiting fragment length polymorphism as compared to SGT-1, was the first to be isolated in the affected area, but may have descended from a different, closely related previous outbreak (Table 2).

**Table 2:** Characterization of the 41 *Bacillus anthracis *outbreaks of the Pollino National Park 2004 epidemic sample

DATE OF OUTBREAK	STRAIN AND SUBGENOTYPE	FARM CODE	LONGITUDE	LATITUDE	ALTITUDE	ANIMAL DEATHS BY SPECIES					TOTAL
							
						BOVINE	OVINE	HORSE	RED DEER	GOAT	
28/07/2004-	069-SGT-5	025PZ072	40.17564	15.96357	1025		1				1

25/08/2004	070/072-SGT-1	097PZ111	39.95204	16.13405	1000	1					1

30/08/2004- I phase	071-SGT-1	034PZ162	40.06903	16.17553	474	4		1			5

30/08/2004- I phase	073-SGT-2 097-SGT-1	097PZ065	40.00476	16.13205	800	9					9

01/09/2004- I phase	075-SGT-1	097PZ190	39.95204	16.13405	1000	1		3			4

02/09/2004- I phase	074-SGT-1	078PZ043	40.04136	16.18231	773	1		2			3

03/09/2004- I phase	078-SGT-1	097PZ009	39.95566	16.11553	800	3					3

05/09/2004- I phase	077-SGT-1	023PZ008	40.03877	15.98671	950	7					7

06/09/2004- I phase	093-SGT-1 081-SGT-1	028PZ007	40.07953	16.16534	458		1				1

06/09/2004- I phase	076-SGT-1	070PZ068	39.975	16.02374	330	8					8

07/09/2004- I phase	087-SGT-1	078PZ004	40.04	16.181	773	7					7

07/09/2004- I phase	083-SGT-1	078PZ012	40.041	16.182	773	7	1				8

07/09/2004- I phase	084-SGT-1	unknown	40.003	16.131	800				1		1

08/09/2004- I phase	092-SGT-1	097PZ107	39.96333	16.07981	620		7				7

09/09/2004- I phase	No isolates	022PZ014	40.02203	16.00367	720	1					1

09/09/2004- I phase	No isolates	022PZ024	40.02101	16.00301	720	1		1			2

09/09/2004- I phase	No isolates	022PZ041	40.01079	16.02706	700	1					1

09/09/2004- I phase	099-SGT-1	022PZ043	40.00578	16.02734	700	3					3

09/09/2004- I phase	No isolates	022PZ106	40.03498	15.99999	900	1					1

09/09/2004- I phase	091-SGT-2	028PZ171	40.08685	16.23801	450	3		1			4

10/09/2004- I phase	098-SGT-1	097PZ105	39.97987	16.16671	950	1					1

10/09/2004- I phase	096-SGT-1	097PZ152	39.99478	16.04499	630		1				1

10/09/2004- I phase	089-SGT-1	unknown	40.06463	16.14047	600				1		1

11/09/2004- I phase	082/095/086-SGT-1	097PZ016	39.95466	16.11453	800		3				3

12/09/2004- I phase	088/094-SGT-1	030PZ027	40.08343	16.07682	636	1					1

13/09/2004- I phase	090-SGT-4	022PZ015	40.01337	15.99796	648				1	4	5

14/09/2004- I phase	085-SGT-1	040PZ014	40.07418	15.99999	600	4	1				5

15/09/2004- I phase	079-SGT-1	042PZ119	unknown	unknown	1300	1				3	4

19/09/2004-II phase	116-SGT-1	040PZ017	40.07418	15.99999	600	1					1

19/09/2004- II phase	080-SGT-1	unknown	40.0242	16.30969	619				1		1

19/09/2004- II phase	117-SGT-1	078PZ096	40.042	16.18589	790	3					3

20/09/2004- II phase	113-SGT-1	022PZ010	40.0206	16.0796	723	1				1	2

21/09/2004- II phase	106/107-SGT-1	031PZ028	40.11861	16.171	620	1				1	2

22/09/2004- II phase	108-SGT-1	042PZ430	40.07374	16.00476	600	4					4

22/09/2004- II phase	No isolates	unknown	40.06388	16.00388	942			3			3

24/09/2004- II phase	112-SGT-3	030PZ003	unknown	unknown	608	1					1

27/09/2004-III phase	118-SGT-1	050PZ064	40.18915	15.90076	988	3					3

27/09/2004- III phase	100/101-SGT-1	unknown	40.04144	16.08247	910				2		2

28/09/2004- III phase	102/103-SGT-1	unknown	40.03934	16.0807	924				1		1

28/09/2004- III phase	114/115-SGT-2	031PZ027	40.11801	16.174	680	1					1

29/09/2004- III phase	111-SGT-1	042PZ080	40.05419	15.86776	936	1					1

03/10/2004- III phase	119-SGT-1	unknown	unknown	unknown	n.a.				1		1

22/09/04	104/105-SGT-1	Isolated from soil	unknown	unknown	n.a.						

21/10/04	120/121-SGT-1	Isolated from feces (wild boar)	unknown	unknown	n.a.						

TOTAL						**81**	**15**	**11**	**9**	**8**	**124**

The 53 *B. anthracis *strains are numbered (from 069 to 121) following the Anthrax Reference Institute of Italy coding system. For each outbreak, the date and phase of the epidemic is reported, as well as the number of animal deaths and their species. The subgenotype and GIS coordinates of the site of sample collection are indicated for each isolated strain.

## Conclusions

The epidemic under study was characterized by a single anthrax clone. Although the epidemic spread over a large area, it involved extremely closely related isolates, 86.7% of which (SGT-1) were identical across the highly discriminating SNR markers. This epidemic was probably exacerbated by the absence of a careful monitoring of animals, making it possible for spores from a recent victim to spread in the environment. The mutational steps found in SGT-2, 3 and 4 may be associated with infective cycles subsequent to the first, while the presence of a more distantly related strain, SGT-5, testifies to the evolutionary history of this lineage in this region. This divergent mutational clone may have originated from different outbreaks in the past. The high throughput genotyping system used in this study proved to be a useful tool for the study of closely related *B. anthracis *strains, and is therefore potentially valuable not only for the study of epidemics, but also for other contexts requiring the characterization of closely related strains such as the study of biological contaminations through environmental isolates or the forensic investigation of bioterrorist events.

## Methods

### Bacillus anthracis isolates

In this study, we analyzed 53 *B. anthracis *strains associated with a single anthrax epidemic.

### DNA preparation

Each *B. anthracis *strain was streaked onto 5% sheep blood agar plates and then incubated at + 37°C for 24 hours. After heat inactivation (98°C for 20 min.), microbial DNA was extracted using DNAeasy Blood and Tissue kit (Qiagen), following the protocol for Gram positive bacteria.

### 25-loci MLVA and SNR analyses

We utilized 5' fluorescent-labelled oligos, deprotected and desalted, specifically selected for the VNTRs and for the SNRs used.

The 25 specific primer pairs for the MLVA were selected as described by Lista *et al*. [17]. The eight specific primer pairs for SNR reactions were selected following Stratilo *et al*. and Kenefic *et al*. [23,24] (Table 3).

**Table 3:** Primers used in this study for MLVA and SNR analyses

	LOCUS	PRIMER SEQUENCE (5' to 3')	CONCENTRATION μM
**MLVA 25**			

	**CG3**	F:CY5.5-TGTCGTTTTACTTCTCTCTCCAATAC R:AGTCATTGTTCTGTATAAAGGGCAT	0.30
	
	**bams44**	F: CY5.5-GCACTTGAATATTTGGCGGTAT R: GCGAATTAATTGCTCCTCAAAT	0.30
	
	**bams3**	F: CY5.5-GCAGCAACAGAAAACTTCTCTCCAATAACA R:TCCTCCCTGAGAACTGCTATCACCTTTAAC	0.30
	
Multiplex A	***vrrB2***	F: D2-CACAGGCTATTCTTTATCAAACTCATC R: CCCAAGGTGAAGATTGTTGTTGA	0.15
	
	**bams5**	F: D2-GCAGGAAGAACAAAAGAAACTAGAAGAGCA R: ATTATTAGCAGGGGCCTCTCCTGCATTACC	0.30
	
	**bams15**	F: D2-GTATTTCCCCCAGATACAGTAATCC R: GTGTACATGTTGATTCATGCTGTTT	0.60
	
	**bams1**	F: CY5-GTTGAGCATGAGAGGTACCTTGTCCTTTTT R: AGTTCAAGCGCCAGAAGGTTATGAGTTATC	0.15
	
	**vrrC1**	F: CY5-GAAGCAAGAAAGTGATGTAGTGGAC R: CATTTCCTCAAGTGCTACAGGTTC	0.30

	**bams13**	F: CY5.5-AATTGAGAAATTGCTGTACCAAACT R: CTAGTGCATTTGACCCTAATCTTGT	0.30
	
	**vrrB1**	F: CY5--ATAGGTGGTTTTCCGCAAGTT R: GATGAGTTTGATAAAGAATAGCCTGTG	0.10
	
Multiplex B	**bams28**	F: CY5-CTCTGTTGTAACAAAATTTCCGTCT R: TATTAAACCAGGCGTTACTTACAGC	0.15
	
	**vrrC2**	F: CY5-CCAGAAGAAGTGGAACCTGTAGCAC R: GTCTTTCCATTAATCGCGCTCTATC	0.10
	
	**bams53**	F: D2-GAGGTGTGTTAGGTGGGCTTAC R: CATATTTTCACCTTAATTTTGGAAG	0.60
	
	**bams31**	F: D2-GCTGTATTTATCGAGCTTCAAAATCT R: GGAGTACTGTTTGTTGAATGTTGTTT	0.60

	**vrrA**	F: CY5.5-CACAACTACCACCGATGGCACA R: GCGCGTTTCGTTTGATTCATAC	0.06
	
	**bams25**	F: CY5.5-CCGAATACGTAAGAAATAAATCCAC R: TGAAAGATCTTGAAAAACAAGCATT	0.15
	
Multiplex C	**bams21**	F: CY5.5-TGTAGTGCCAGATTTGTCTTCTGTA R: CAAATTTTGAGATGGGAGTTTTACT	0.30
	
	**bams34**	F: D2-TGTGCTAAATCATCTTGCTTGG R: CAGCAAAATCAATCGAATCAAA	0.30
	
	**bams24**	F: CY5-CTTCTACTTCCGTACTTGAAATTGG R: CGTCACGTACCATTTAATGTTGTTA	0.30

	**bams51**	F: CY5-ATTTCCTGAAGCAGGTTGTGTT R: TGCATCTAACAATGCAGAACAA	0.60
	
	**bams22**	F: CY5-ATCAAAAATTCTTGGCAGACTGA R: ACCGTTAATTCACGTTTAGCAGA	0.15
	
Multiplex D	**bams23**	F: D2-CGGTCTGTCTCTATTATTCAGTGGT R: CCTGTTGCTCCTAGTGATTTCTTAC	0.30
	
	**bams30**	F: CY5.5-AGCTAATCACCTACAACACCTGGTA R: CAGAAAATATTGGACCTACCTTCC	0.30
	
	**pXO1**	F: CY5-CAATTTATTAACGATCAGATTAAGTTCA R: TCTAGAATTAGTTGCTTCATAATGGC	0.15
	
	**pXO2**	F: CY5.5-TCATCCTCTTTTAAGTCTTGGGT R: GTGTGATGAACTCCGACGACA	0.15

**STR-MTD**			

Singleplex A	**CL33**	F: 6FAM-TGGGGTATATTCCCATCGAA R:CCGCAGATACCAACCAACAT	0.2

Singleplex B	**CL12**	F: 6FAM-AAGCCAGGTGCAAAAACAGT R: TCTCACTGTGCCTCGCTAAA	0.2

Singleplex C	**CL1**	F: 6FAM-TTCTCGGAGATGATTTTCGG R:CTCCCATTTTACATCCCCCT	0.2

Singleplex D	**CL37**	F: NED-CTCCGCAATTTTCAAACGAT R: CCGCCGGCATAAAGATAGTA	0.2

**KEN-MTD**			

	**CL33** **HM1**	F: PET GAAAACTTTGCAACCGACC R: GTCGAACGTGTTCTAGCTACAG	0.2
	
Multiplex I	**CL10** **HM6**	F: 6FAM-TAAAAAGACAGAATTTTCAATTTTATCAACAAC R: GTGGAAACTAATGTGAGTTATATATGTTAGTTAAG	0.2
	
	**CL12** **HM2**	F: VIC-GCTATTCTCACTGTGCCTCG R: GTTAAAACGAAGTAAAGAAAAGTGGG	0.2
	
	**CL35** **HM13**	F: NED-GGATTGCTTAAGGTATATAATGGATTT R: GTTGTGTTCCATATGTATCCCTCC	0.1

MLVA PCRs were performed in four multiplex reactions in a final volume of 15 μl. The reaction mixture contained: 1× PCR reaction buffer (Roche), 1 U of Taq DNA polymerase (Roche), dNTPs (0.2 mM each), and appropriate concentrations of each primer as reported in Table 3. The thermocycling conditions were as follows: 96°C for 3 min; 36 cycles at 95°C for 20 s, at 60°C for 30 s, and at 72°C for 1 min; and finally, 72°C for 10 min.

The KEN-MTD PCR was performed in a multiplex reaction in a final volume of 25 μl containing 1× AmpliTaq Gold PCR buffer and 0.5 U of AmpliTaq Gold DNA polymerase (Applied Biosystems Inc.), 3,5 mM MgCl_2_, dNTPs (0.2 mM each), and appropriate concentrations of forward and reverse primers as reported in Table 3.

STR-MTD PCRs were performed in four singleplex reactions in a final volume of 25 μl containing 1× AmpliTaq Gold PCR buffer and 0.5 U of AmpliTaq Gold DNA polymerase (Applied Biosystems Inc.), 4 mM MgCl_2_, dNTPs (0.2 mM each), and appropriate concentrations of forward and reverse primers as reported in Table 3. The thermocycling conditions were as follows: 95°C for 5 min; 35 cycles at 94°C for 30 s, 60°C for 30 s, and 72°C for 30 s; and finally, 72°C for 7 min.

### Automated genotype analysis

MLVA PCR products were diluted 1:5. Five μl of solution were added to a mix containing 40 μl of Sample Loading Solution (Beckman Coulter) and 0.5 μl of MapMarker 1000 size marker (BioVentures Inc.). Amplicons were separated by electrophoresis on a CEQ 8000 automated DNA Analysis System (Beckman Coulter) and sized by CEQ Fragment Analysis System software.

Amplified SNR PCR products were diluted 1:80 and subjected to capillary electrophoresis on ABI Prism 3130 genetic analyzer (Applied Biosystems) with 0.25 μl of GeneScan 120 Liz and 500 Liz size standards for KEN-MTD and STR-MTD amplicons, respectively, and sized by GeneMapper 4.0 (Applied Biosystems Inc.). DNA extracted from each sample was tested by two different laboratories: the Anthrax Reference Institute of Italy, and the Army Medical and Veterinary Research Institute.

## Authors' contributions

GG conceived the study, participated in its design, coordinated the molecular tests and drafted the manuscript. AC participated in performing the 25-loci MLVA and the SNR analyses and helped to draft the manuscript. AF designed the study and coordinated the collection of samples. SS performed the SNR analysis and helped draft the manuscript. VP participated in performing the 25-loci MLVA. RA helped design the study. FL coordinated the work of the two laboratories and revised the final version of the manuscript. All authors read and approved the final manuscript.
